# Evaluation of The Effect of Nd: YAG and CO2 Laser Dentin Treatment Techniques on Shear Bond Strength of Composite Resin -

**DOI:** 10.31661/gmj.vi.3863

**Published:** 2025-11-08

**Authors:** Farahnaz Sharafeddin, Arefeh Torabi Parizi, Maryam Jamshidi

**Affiliations:** ^1^ Department of Operative Dentistry, Biomaterials Research Center, School of Dentistry, Shiraz University of Medical Sciences, Shiraz, Iran; ^2^ Department of Operative Dentistry, School of Dentistry, Shiraz University of Medical Sciences, Shiraz, Iran; ^3^ Department of Operative Dentistry, School of Dentistry, Shiraz University of Medical Sciences, Shiraz, Iran

**Keywords:** Nd-YAG Laser, CO2 Lasers, Shear Strength, Composite resins

## Abstract

**Background:**

Recently, lasers have been used for composite resin adhesion, yielding conflicting results. The shear bond strength (SBS) of resin composites to dentin when lasers are used as an alternative to acid etching or in conjunction with acid etching remains unclear. This study aimed to investigate the effect of Nd: YAG and CO2 laser as treatment techniques on composite resin bond strength.

**Materials and Methods:**

Seventy teeth were divided into seven groups: G1 (control) received 37% phosphoric acid etching, G2 was treated with a CO2 laser, G3 with an Nd: YAG laser, G4 with a combination of CO2 and Nd: YAG lasers, G5 underwent CO2 laser treatment after acid etching, G6 received Nd: YAG laser treatment after acid etching, and G7 underwent a combination of CO2 and Nd: YAG lasers after acid etching. Adper Single Bond 2 was used. SBS was determined after thermocycling and statistically analyzed using the Kruskal-Wallis test. Specimens were examined for fracture types under a stereomicroscope at 40X magnification.

**Results:**

Control group had highest SBS (11.14±1.97 MPa, p≤0.004 vs all). Acid+Nd:YAG (8.19±3.13 MPa) showed better bonding than CO2 (2.07±1.38, P=0.001), Nd:YAG (3.11±2.11, P=0.002), and Acid+CO2 (2.38±1.52, P=0.001). Adhesive failures dominated (73%); no cohesive failures occurred. Acid+Nd: YAG performed best among surface treatments.

**Conclusion:**

The SBS decreased following the application of CO2 and Nd: YAG lasers, either alone or in combination. However, after the application of phosphoric acid, lasers yielded improved results, although they remained lower than the control group.

## Introduction

Dental composite resin repairs decayed teeth with a tooth-colored aesthetic alternative to amalgam. Its resin matrix and silica fillers enhance strength and durability while reducing wear [1]. Adhesion of dental restorative materials to dentin is more challenging than adhesion to enamel due to its lower mineral content and higher water content [2][3]. Achieving strong and durable adhesion in restorative dentistry relies on creating a stable hybrid layer, the fundamental principle of optimal bonding to dentin. The hybrid layer, also known as the resin-dentin interfusion zone, results from the penetration of resin monomers into the exposed intertubular dentin and collagen network following demineralization [3]. This process is critical for long-term restoration success, as it mechanically locks the restorative material to the tooth. The formation of the hybrid layer is influenced by various factors, including the quality of the dentin substrate, the type of adhesive system, and tooth pretreatment. Proper dentin conditioning is a fundamental principle for composite restoration in dentin defects and ensures the success of the restoration [2][3].

In conventional adhesive dentistry, the gold standard for adhesion is the use of phosphoric acid to etch the dentin substrate [4]. However, this method has drawbacks, such as the risk of the acid reaching healthy tooth tissue, pulp irritation, and increased sensitivity. These limitations have driven the search for alternative techniques. The presence of these issues, along with the quest to enhance the bond strength between resin and dentin while strengthening the hybrid layer, has led researchers to explore new techniques, including the use of lasers to treat dentin surfaces [5][6].

Emerging evidence suggests that lasers, such as Neodymium-Doped Yttrium Aluminum Garnet (Nd: YAG) lasers and Carbon dioxide (CO2) lasers, could improve dentin bonding by removing the smear layer and opening the dentin tubules. Additionally, lasers can sterilize the dentin surface and create micron-sized porosities that facilitate better penetration of resin monomers, resulting in the formation of a stable hybrid layer [7].

Morphological studies using scanning electron microscopy have revealed irregularities, such as honeycomb or crater-like structures, in dentin and enamel after the use of Nd: YAG and CO2 lasers [8]. The effect of laser treatment creates a rough dentin surface, which theoretically provides more micromechanical retention and enhances bond strength [8][9][10]. However, conflicting findings exist, as CO2 and Nd: YAG lasers could reduce bond strength by causing fusion, melting, and recrystallization of the dentin structure [8][11]. The specific impact of these laser effects depends on various factors, including the laser's parameters and the quality of the dentin substrate [12]. Given these complexities, further studies are necessary to determine the precise influence of lasers on bond strength. To address these knowledge gaps, the present study investigates the effect of Nd:YAG and CO2 lasers, both individually and in combination, with or without prior acid etching, on the shear bond strength (SBS) of resin composite to dentin. The null hypotheses of this study are as follows: 1) CO2 or Nd:YAG lasers do not affect the bond strength between resin and dentin; 2) The simultaneous use of Nd:YAG and CO2 lasers does not impact the SBS of resin to dentin; 3) The application of Nd:YAG and CO2 lasers, either separately or together after acid etching, does not influence the SBS of resin to dentin.

## Materials and Methods

This was an experimental study conducted in 2024 at dental department of shiraz university of medical sciences and study received ethical approval with code of IR.SUMS.DENTAL.REC.1399.130. Seventy caries-free, intact human molars, extracted for periodontal reasons (not for this study), were collected. These teeth had been extracted due to periodontal issues and were not obtained specifically for this research. The teeth were collected one month before the commencement of the study and were stored in a 0.1% thymol solution (pH=7) at 4°C in a refrigerator during this period. Prior to the study, the teeth were rinsed under running water for 60 s.

Subsequently, the teeth were cervically embedded in self-curing acrylic resin (Acropars, Marlic Medical Co., Tehran, Iran), creating specimens measuring 2 cm (length) × 1.5 cm (height) × 2 cm (width). The embedding was carried out until the cementoenamel junction (CEJ) was level with the acrylic resin surface, ensuring that the occlusal surfaces were parallel to the acrylic resin surface. After polymerization, the teeth were sectioned to a depth of 0.5 mm below the dentin-enamel junction (DEJ) of the occlusal surface using a diamond disc (D & Z, Germany). This procedure was performed with water cooling to achieve a smooth dentin surface, followed by polishing using 600-grit silicon carbide (SiC) paper (Piramit, Istanbul, Turkey) to homogenize the surface.

The samples were then randomly divided into seven groups (n=10 per group) using a simple randomization method. Each group underwent in vitro shear bond strength (SBS) testing.

• Group 1 (Control): After 15 s of etching with 37% phosphoric acid (Denfil, Vericom, Korea), the dentin surface was rinsed with a water spray from a distance of 1 cm for 20 s. Excess water was removed using cotton pellets.

• Group 2: A CO2 laser (DEKA, Smartxide, Italy) was applied for 60 s in a circular pattern (3 mm diameter) at the center of the sample. The CO2 Surgical Laser Handpiece tip (300 μm diameter) was held perpendicular to the sample’s surface at a distance of 1 mm.

• Group 3: An Nd:YAG laser (Fotona LightWalker AT/AT S) with a fiber optic handpiece (Model R21-C2, 200 μm diameter) was used in non-contact mode for 60 s on the tooth’s surface. The laser tip was held perpendicular to the sample at a distance of 1 mm.

• Group 4: CO2 laser (60 s) was applied, followed by Nd:YAG laser (60 s) on the same surface. Both lasers were held perpendicular to the sample at 1 mm distance.

• Group 5: After 15 s of etching with 37% phosphoric acid, CO2 laser (60 s) was applied in a 3 mm circular pattern. The CO2 Surgical Laser Handpiece tip was held perpendicular at 1 mm distance.

• Group 6: After 15 s of etching with 37% phosphoric acid, Nd: YAG laser (60 s) was applied using the R21-C2 handpiece, held perpendicular at 1 mm distance.

• Group 7: After 15 s of etching with 37% phosphoric acid, CO2 laser (60 s) followed by Nd: YAG laser (60 s) were applied, both held perpendicular at 1 mm distance.

Following laser treatments, the bonding system (Adper Single Bond 2, 3M ESPE, USA) was actively applied to the dentin in two layers using a microbrush for 15 s, followed by a 5-s gentle air blast to evaporate solvents. The adhesive was then cured for 10 s using an LED curing light (Demi Plus, Kerr, Switzerland; intensity: 1200 mW/cm², wavelength: 470 nm).

A nano-hybrid composite (Z350, Dentin A2, 3M ESPE, USA) was placed on the dentin using a Teflon mold (3 mm diameter × 2 mm height) and cured for 40 s with the LED device. The samples were incubated in distilled water at 37°C (Nuve ES250 Cooled Incubator, Turkey) for 24 h.

**Figure-1:**
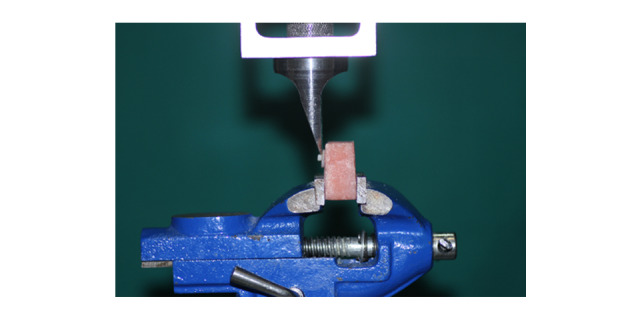
Figure 1. A prepared sample in the universal testing machine

Shear bond strength (SBS) testing was performed using a knife-edge blade at a crosshead speed of 1 mm/min until failure occurred (Figure-1). A stereomicroscope (BestScope BS-3060C, China; 40× magnification) was used to examine fracture types. Two blinded observers, unaware of the restorative treatments, classified fractures as:

1. Cohesive Type I: Within the composite resin.

2. Cohesive Type II: Within dentin.

3. Adhesive: At the dentin/adhesive or adhesive/resin interfaces.

4. Mixed: Combination of cohesive and adhesive fractures.

Statistical analysis was conducted using the Kruskal-Wallis test due to non-parametric nature of data (IBM SPSS Statistics v22.0). The significance level was set at P<0.05.

## Results

The Kruskal-Wallis test revealed statistically significant differences in shear bond strength (SBS) values among the experimental groups (P<0.05).

Post hoc pairwise comparisons with adjusted P-values indicated that the control group (11.14 ± 1.97 MPa) exhibited significantly higher SBS values compared to all other groups (P≤0.004).

**Table:** Table1. Materials

Material	composition
resin composite: Nano hybrid Filtek™ Z350 XT A1 shade(3M ESPE USA)	Resin Matrix: Bis-GMA, UDMA, TEGDMA, Dimethacrylate Filler content: 78.5wt% (59.5 vol%) Silica, zirconia, aggregated zirconia/silica
Adhesive system: single bond2(3M ESPE USA adper)	HEMA, bis-GMA, ethyl alcohol, silane-treated silica (nanofiller), glycerol 1,3-dimethacrylate, copolymer of acrylic and itaconic acids, diurethane dimethacrylate, water, 10% by weight of silica nanoparticles
Nd:YAG laser(Fotona LSN001298,Finlnd)	wavelength:1064nm power of 1W energy:100 mj pulse repetition:10HZ pulse duration: 60 s spot diameter at the tissue: circle with a diameter of 3 mm
CO2 laser(DEKA smart US.20)	wave length:10.6 nm power of 2 W CW single mode Pulse duration: 60 s spot diameter at the tissue: circle with a diameter of 3 mm

Additionally, the Acid+Nd: YAG group (8.19 ± 3.13 MPa) demonstrated significantly greater SBS than the CO2 (2.07 ± 1.38 MPa; P=0.001), Nd: YAG (3.11 ± 2.11 MPa; P=0.002), CO2+Nd: YAG (1.76 ± 1.24 MPa; P=0.001), Acid+CO2 (2.38 ± 1.52 MPa; P=0.001), and Acid+CO2+Nd: YAG (5.82 ± 2.66 MPa; P=0.104) groups, except for the latter, which showed no significant difference (P=0.104), as shown in Table-2.

**Table:** Table2. Mean ± SD of SBS Values (MPa) of Different Experimental Groups

Groups	Mean ± SD
Control	11.14 ± 1.97
CO2	2.07 ± 1.38
Nd:YAG	3.11 ± 2.11
CO2+Nd:YAG	1.76 ± 1.24
Acid+CO2	2.38 ± 1.52
Acid+Nd:YAG	8.19 ± 3.13
Acid+CO2+Nd:YAG	5.82 ± 2.66

**Table:** Table3. Fracture Mode of the Study Groups

Groups	Adhesive	Cohesive in composite	Cohesive in dentin	Mix
Control	4	__	__	6
CO2	8	__	__	2
Nd:YAG	7	__	__	3
CO2+Nd:YAG	7	__	__	3
Acid+CO2	9	__	__	1
Acid+Nd:YAG	8	__	__	2
Acid+CO2+Nd:YAG	8	__	__	2

Further comparisons revealed that the CO2 and CO2+Nd: YAG groups did not differ significantly (P=0.940), nor did the CO2 and Acid+CO2 groups (P=0.545) or the Nd: YAG and Acid+CO2 groups (P=0.427).

**Figure-2:**
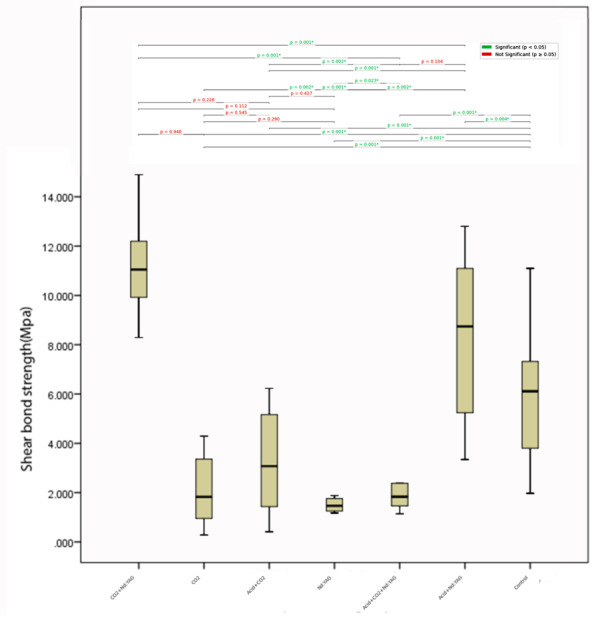
Figure 2. Pairwise comparison of different groups

However, the Acid+CO2+Nd: YAG group had significantly lower SBS than the control (P=0.001) but higher than the CO2 (P=0.002) and Acid+CO2 (P=0.002) groups. These findings suggest that surface treatments involving laser etching (CO2, Nd: YAG, or combined) and acid application significantly reduce SBS compared to untreated enamel, with the Acid+Nd: YAG group showing the highest bond strength among treated surfaces. Figure-2 illustrates the results of the SBS comparisons between different groups.

**Figure-3:**
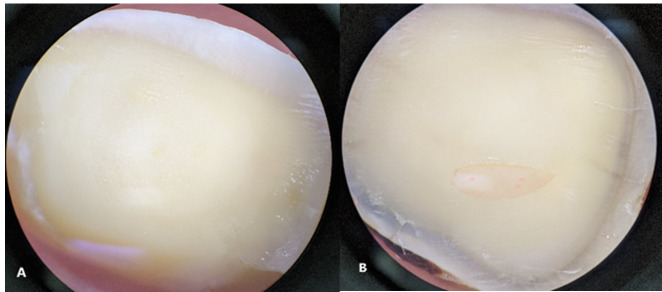
Figure 3. Types of fracture mode: (A) Adhesive failure, (B) Mixed failure

Table 4 presents the results related to the fracture modes in the studied groups.

The failure mode distribution across different experimental groups was reported as follows: Control (adhesive=4, mix=6), CO2 (adhesive=8, mix=2), Nd: YAG (adhesive=7, mix=3), CO2 + Nd: YAG (adhesive=7, mix=3), Acid + CO2 (adhesive=9, mix=1), Acid + Nd: YAG (adhesive=8, mix=2), and Acid + CO2 + Nd: YAG (adhesive=8, mix=2), with no observed cohesive failures in composite or dentin for any group.

Adhesive failure was the most common type, accounting for 73% of all failures, followed by mixed failure, which accounted for 27% of all failures.

## Discussion

To achieve a durable bond between dentin and composite resin, the adhesive must penetrate the exposed collagen network and establish a stable hybrid layer at the dentin-resin interface. Dentin, particularly deep dentin, is inherently moist, which can interfere with full resin penetration into the exposed collagen network [14].

The results of our present study indicate that treating the dentin surface with an Nd: YAG laser, with or without acid etching, reduces the SBS of the resin to the tooth. This finding aligns with a study conducted by T.M. Silva et al., who utilized an Nd: YAG laser with a wavelength of 1064 nm, a frequency of 10 Hz, and various energy parameters in their design. They demonstrated that using an Nd: YAG laser prior to applying the adhesive system reduced micro-tensile bond strength. This reduction resulted from the degradation of the organic dentin content due to laser-generated heat and the occlusion of dentin tubules due to the fusion and recrystallization of inorganic content [15].

In another study, the effects of Nd: YAG laser were investigated before and after the application of Single Bond 2. They observed that when the laser was used before bonding as a substitute for phosphoric acid, tensile bond strength decreased, which is consistent with our present study's findings. The reason for this decrease appears to be the complete or partial closure of the tubules and the recrystallization of dentin components. However, when the laser was applied after the adhesive and before its polymerization, tensile bond strength increased due to improved resin monomer penetration and the formation of a stable hybrid layer [16].

The timing of laser application in relation to adhesive application significantly influences the results of various bond strength tests. In our study, the Nd: YAG laser seems to seal the dentinal tubules when applied before the adhesive, thus reducing SBS, as observed in the aforementioned study.

Marimoto et al. reported an increase in resin bond strength to the tooth after Nd: YAG laser application [17]. In contrast, our study found the opposite effect. The discrepancy appears to lie in the timing of laser application. In their study, the laser was applied after bonding, which increased resin monomer penetration. This occurred because the dentin tubules had not yet closed due to the laser's heat, which also lowered resin viscosity, enhancing penetration. However, when the laser was used before applying the resin in our study, the heat caused tubules to fuse, preventing proper resin penetration into the dentin tubules.

Another study examined the effect of Nd: YAG laser on self-etch and two-step etch-and-rinse adhesives. They reported improved bond strength for self-etch bonding but found no difference with etch-and-rinse adhesive. Their study revealed that laser use increased the concentration of calcium and phosphorus in dentin. Some monomers, such as 10-MDP and 4META, can chemically bond with calcium hydroxyapatite. The increased chemical bond between the acidic groups in these adhesives and the elevated calcium content could be responsible for the improved bond strength of the two self-etch adhesives [18]. In our study, Single Bond 2, a two-step etch-and-rinse adhesive, was used. As this adhesive lacks 10-MDP and 4META monomers, the difference in composition may explain the decrease in SBS.

According to the results of our study, SBS decreased after using the CO2 laser. Rechmann P et al. also observed a reduction in SBS after CO2 laser application. This reduction was up to 60% for the fifth generation of adhesive systems compared to the control group that did not receive laser treatment. The melting of the dentin surface due to the heat generated by the laser, which causes incomplete adhesive penetration, may be responsible for the reduction in SBS [19]

. Additionally, the CO2 laser has a high absorption coefficient in water and organic materials, enabling it to remove the smear layer, close dentin tubules, and alter dentin permeability [20]. The decrease in bond strength in our study may be attributed to the incomplete removal of the smear layer, insufficient resin monomer penetration into fully or partially blocked dentinal tubules, and the disruption of the collagen network.

In a study by Ding M et al., it was shown that if etching with phosphoric acid is used after CO2 laser, the bond strength will decrease. They stated that the laser alters the dentin structure and produces etch-resistant structures that prevent the penetration of monomers and also impair mechanical adhesion [21]. From the results of our study, it seems that the laser has changed the structure of dentin in such a way that whether used alone or with phosphoric acid, optimal resin monomer penetration into the collagen network is hindered, preventing the formation of an ideal hybrid layer.

Until now, no study had investigated the simultaneous effect of Nd: YAG and CO2 lasers on the SBS of resin composite to dentin. This study aimed to explore this simultaneous effect. It was observed that the simultaneous application of these two lasers reduced the SBS, leading to the rejection of the second null hypothesis. Initially, it was hypothesized that the concurrent use of two lasers might create more irregularities, enhancing composite resin retention. However, the results showed that the simultaneous laser application had a more significant negative impact on the SBS of dentin resin. This reduction in SBS may be attributed to the closure of more tubules, complete closure of each tubule, and further destruction of collagen fibrils, despite the formation of irregularities. The Nd: YAG laser seemed to intensify the CO2 laser's effect, generating more heat during dentin conditioning. This increase in temperature likely led to more dentin tubule occlusion, denaturation of the collagen network, and disruption of the hybrid layer. As a result, resin monomer penetration into the collagen network and dentin tubules likely decreased, leading to the reduced SBS.

In a separate study, the effect of pulsed Nd: YAG laser on the tensile bond strength of resin to enamel was investigated as an alternative to acid etching, both after and before acid etching. The use of Nd: YAG laser after acid etching did not significantly differ from using the laser alone, and both were less effective than the acid etching group. The highest tensile bond strength was observed when phosphoric acid was used after laser treatment [22]. In our study, laser application after acid etching yielded superior results compared to cases without acid etching. A similar study by Davari et al. reported comparable findings. In their research, the effect of Er: YAG laser on the SBS of composite to dentin was investigated. They found that although the SBS using Er: YAG laser after acid etching was slightly higher than that of the acid-etched group, the differences were not statistically significant [23]. In our study, Nd: YAG laser after acid etching resulted in higher SBS than cases without acid etching. Furthermore, the simultaneous application of Nd: YAG and CO2 lasers after acid etching produced better results than cases without acid etching. It appears that using the laser after acid etching prepares the dentin surface more effectively to receive the adhesive. The improved porosities created on acid-etched dentin by laser etching, along with the irregularities produced by acid etching and the additional surface roughness from laser pretreatment, likely contributed to this effect. However, in our study, the SBS of laser application after acid etching was still lower than that of acid etching with phosphoric acid alone.

Upon examination using a stereo microscope, adhesive failure was found to be the most common failure in all groups. This suggests that the adhesive is the weakest link in bonding, and stress concentration is high at the adhesive-composite interface. Since the application of laser does not promote the formation of a suitable hybrid layer, adhesive failure is the most common type of failure, as proper bonding has not been established. No cohesive failure was observed in the composite, indicating that the SBS test was well conducted, and there was no induction of stress in unwanted areas.

In the future, it is suggested to investigate the simultaneous use of lasers after the application of the adhesive and before its polymerization. This may facilitate deeper penetration of resin monomers into the collagen network, thereby potentially increasing bond strength. Additionally, it is recommended to explore the combined effects of two lasers on self-etch adhesives containing 10-MDP monomers. These adhesives may exhibit improved bonding to laser-treated surfaces compared to the total etch adhesives used in this study.

## Conclusion

The results of this study indicate that the use of CO2 and Nd:YAG lasers, as well as their simultaneous application on dentin using Single Bond 2 adhesive, reduces the SBS of the resin to dentin compared to the application of 37% phosphoric acid. However, after the application of phosphoric acid, lasers yielded better results, although they still fell short of the bond strength achieved with acid etching. Therefore, further investigation is needed to assess the viability of replacing phosphoric acid with lasers to enhance bond strength.

## Conflict of Interest

The authors declare no conflicts of interest.
